# Supplementary material for: Effects of both Pro- and Synbiotics in Liver Surgery and Transplantation with Special Focus on the Gut–Liver Axis—A Systematic Review and Meta-Analysis
Source: Nutrients. 2020 Aug 15;12(8):2461. doi: 10.3390/nu12082461 (PMC7468972; doi:10.3390/nu12082461)
Supplement: Supplementary file 1 [file nutrients-12-02461-s001.pdf]

File S1: Search Strategies

Search PubMed - 30.06.2020

#1 Search: probiotic OR probiot* OR synbiotic* OR symbiotic - 28,940 Treffer

#2 Search: operation* OR surgical procedure* OR liver surgery OR liver surg* OR liver transplantation* OR hepatectomy OR liver resection OR liver resect* - 1,506,214 Treffer

#3 Search: mortality OR morbidity OR sepsis OR surgical infection* OR surgery site infection* OR post-operative wound infection* OR postoperative wound infection* OR complication* - 5,737,725 Treffer

#4 Search: ((probiotic OR probiot* OR synbiotic* OR synbiotic) AND (operation* OR surgical procedure* OR liver surgery OR liver surg* OR liver transplantation* OR hepatectomy OR liver resection OR liver resect*)) AND (mortality OR morbidity OR sepsis OR surgical infection* OR surgery site infection* OR post-operative wound infection* OR postoperative wound infection* OR complication*) - 290 Treffer
